# Smartwatch-Detected Arrhythmias in Patients After Transcatheter Aortic Valve Replacement (TAVR): Analysis of the SMART TAVR Trial

**DOI:** 10.2196/41843

**Published:** 2024-07-19

**Authors:** Jiaqi Fan, Hanyi Dai, Yuchao Guo, Jianguo Xu, Lihan Wang, Jubo Jiang, Xinping Lin, Cheng Li, Dao Zhou, Huajun Li, Xianbao Liu, Jian'an Wang

**Affiliations:** 1 department of Cardiology Zhejiang University Hangzhou China

**Keywords:** arrhythmias, transcatheter aortic valve replacement, smartwatch, ambulatory electrocardiography, smartphone, mobile phone

## Abstract

**Background:**

There are limited data available on the development of arrhythmias in patients at risk of high-degree atrioventricular block (HAVB) or complete heart block (CHB) following transcatheter aortic valve replacement (TAVR).

**Objective:**

This study aimed to explore the incidence and evolution of arrhythmias by monitoring patients at risk of HAVB or CHB after TAVR using smartwatches.

**Methods:**

We analyzed 188 consecutive patients in the prospective SMART TAVR (smartwatch-facilitated early discharge in patients undergoing TAVR) trial. Patients were divided into 2 groups according to the risk of HAVB or CHB. Patients were required to trigger a single-lead electrocardiogram (ECG) recording and send it to the Heart Health App via their smartphone. Physicians in the central ECG core lab would then analyze the ECG. The incidence and timing of arrhythmias and pacemaker implantation within a 30-day follow-up were compared. All arrhythmic events were adjudicated in a central ECG core lab.

**Results:**

The mean age of the patients was 73.1 (SD 7.3) years, of whom 105 (55.9%) were men. The mean discharge day after TAVR was 2.0 (SD 1.8) days. There were no statistically significant changes in the evolution of atrial fibrillation or atrial flutter, Mobitz I, Mobitz II, and third-degree atrial ventricular block over time in the first month after TAVR. The incidence of the left bundle branch block (LBBB) increased in the first week and decreased in the subsequent 3 weeks significantly (*P*<.001). Patients at higher risk of HAVB or CHB received more pacemaker implantation after discharge (n=8, 9.6% vs n=2, 1.9%; *P*=.04). The incidence of LBBB was higher in the group with higher HAVB or CHB risk (n=47, 56.6% vs n=34, 32.4%; *P*=.001). The independent predictors for pacemaker implantation were age, baseline atrial fibrillation, baseline right bundle branch block, Mobitz II, and third-degree atrioventricular block detected by the smartwatch.

**Conclusions:**

Except for LBBB, no change in arrhythmias was observed over time in the first month after TAVR. A higher incidence of pacemaker implantation after discharge was observed in patients at risk of HAVB or CHB. However, Mobitz II and third-degree atrioventricular block detected by the smartwatch during follow-ups were more valuable indicators to predict pacemaker implantation after discharge from the index TAVR.

**Trial Registration:**

ClinicalTrials.gov NCT04454177; https://clinicaltrials.gov/study/NCT04454177

## Introduction

Transcatheter aortic valve replacement (TAVR) has been established as a preferred treatment for patients with symptomatic severe aortic stenosis with overall risk profiles. However, there are some drawbacks to this procedure, such as the new-onset rhythm and conduction disturbance (CD). The management of arrhythmias and CDs is still challenging even with tailored postoperative management, according to the recent scientific expert panel [1,2]. The scientific expert panel proposed an algorithm based on baseline and post-TAVR electrocardiography within the index hospitalization [1]. Patients were categorized into groups suitable for early discharge (within 48 hours after TAVR), groups at high risk of high-degree atrioventricular block (HAVB) or complete heart block (CHB), and groups in need of pacemaker implantation before discharge [1]. However, this tailored prespecified algorithm falls short in accurately identifying patients at high risk of HAVB or CHB [2]. Therefore, the adoption of ambulatory electrocardiogram (ECG) monitoring in a specific subset of these patients will enhance patient-centered management after discharge. The 2019 algorithm recommends using 30-day ambulatory ECG monitoring in TAVR recipients [1]. Recently, 2 studies using the patch and implantable loop recorders observed a considerable rate of delayed HAVB or CHB after TAVR [3,4]. A smartwatch, a type of ambulatory ECG monitoring, enables single-lead ECG checks after activation by patients, showing enough efficacy and safety in patients who had a TAVR [5]. To date, no study has evaluated the incidence and evolution of arrhythmias and CDs after TAVR using a noninvasive and convenient smartwatch.

In this study, we aim to explore the incidence and evolution of arrhythmias and CDs detected by smartwatches over time in patients at high risk of HAVB or CHB following TAVR within a 30-day follow-up.

## Methods

This was a single-center, prospective trial, called the SMART TAVR (smartwatch-facilitated early discharge in patients undergoing TAVR) trial, registered with the ClinicalTrials.gov registry (NCT04454177).

### Ethical Considerations

Informed consent was provided by all patients to participate in the study before the procedures. The protocol was approved by the medical ethics committee of the Second Affiliated Hospital of Zhejiang University (IR2020001223). This study was conducted according to the Declaration of Helsinki and the Good Clinical Practice guidelines. The data were anonymous and deidentified for every participant.

### Patient Population

Patients with symptomatic severe aortic stenosis who had received the elective transfemoral TAVR in our hospital were consecutively included in the study. Severe aortic stenosis was defined as a median transvalvular gradient of more than 40 mmHg, a peak jet velocity greater than 4 meters per second, or an initial aortic valve area less than 1 cm^2^. Exclusion criteria included severe complications of TAVR (eg, death, conversion to surgical aortic valve replacement, or unplanned cardiopulmonary resuscitation) during the procedure, life expectancy less than 12 months, severe dementia (ie, unable to sign for informed consent, take care of themselves, or communicate with the research team during a study visit). In addition, patients with pacemaker implantation at baseline and predischarge were also excluded.

Participants were classified into groups of high and low risk of HAVB or CHB based on our conduction management protocol in ECG. The incidence and time of arrhythmias and pacemaker implantation within a 30-day follow-up were compared.

### Algorithm for Patients at High Risk of HAVB or CHB

Twelve-lead ECGs were recorded and analyzed at baseline, immediate postoperative period, 4 hours and 24 hours after the procedure, on a daily basis, and thereafter, during the index hospitalization, if needed. Higher risk of HAVB or CHB was defined as the occurrence of at least one of the following: (1) increased PR or QRS interval ≥20 milliseconds within the last two 12-lead ECGs before discharge; (2) PR ≥240 millisecond or QRS ≥150 milliseconds (QRS ≥140 ms for patients with atrial fibrillation) in the last 12-lead ECG before discharge; and (3) transient or persisted HAVB or CHB occurring within the 12-lead ECGs mentioned above. Our CD management protocol was described in detail in the supplementary methods in Multimedia Appendix 1. This protocol was modified based on the previously published Expert Consensus Algorithm for the management of CDs in 2019 [1].

### Smartwatch Equipment and Monitoring Frequency

The enrolled patients were provided with a smartwatch within 24 hours before the scheduled TAVR procedure and were required not to share their smartwatches with anyone else. HUAWEI Watch GT series could continuously monitor and record multiple biometric parameters, including heart rate, step counts, sleep cycles, RR interval, and pulse oxygen saturation. Moreover, these devices could record the single-lead ECG upon activation and analyze QRS complexes and P waves. Patients were required to activate the watch to record ECG twice per day in the week following TAVR discharge and at least 2 days a week for the rest of the month, as described in a previous study [6]. The duration of the recording was at least 30 seconds, and the recordings were applied in the morning and afternoon each day. Moreover, patients were required to upload smartwatch readings at the onset of any cardiovascular symptoms, including dyspnea, chest pain, palpitations, dizziness, or presyncope. The data recorded by HUAWEI Watch were transmitted to the HUAWEI phone app. The Heart Health App (developed by Second Affiliated Hospital of Zhejiang University) would then receive, process, and store the data and transfer it to the remote database after securing patients’ approval. A designated heart team member would access and download the data via a cloud database. All arrhythmic events were adjudicated in a central ECG core lab.

### Data Collection and Definitions

Baseline clinical, electrocardiographic, imaging, and periprocedural characteristics data were collected. The single-lead ECG recording arrhythmias were detected by the smartwatch and evaluated by the central ECG core lab. A persistent left bundle branch block (LBBB) was defined as an LBBB that persisted during the whole 30-day follow-up period. All living patients were assessed face to face in the 30-day follow-up. All TAVR-related variables and outcomes were defined according to the Valve Academic Research Consortium-2 criteria [7]. Patients were categorized into a group at higher risk of HAVB or CHB and a group at lower risk of HAVB or CHB who were eligible for next-day discharge. Variables and outcomes were compared between the two groups. All data were stored in the TORCH registry and SMART TAVR registry databases and were trackable.

### Statistical Analysis

Continuous variables were presented as median (SD) values. Categorical data were shown as counts (percentages). Student *t* test (2-tailed), Mann-Whitney *U* test, or Kruskal-Wallis test were used for continuous variables, while chi-square or Fisher exact test were used for categorical data. Predictors of pacemaker implantation were assessed by Cox regression models. Variables with *P*<.1 in the univariate analysis and commonly reported predictor factors in previous studies were included in the multivariate stepwise regression model. Kaplan-Meier estimates were calculated to describe the probability of pacemaker implantation with the date of TAVR as a starting point according to the risk of HAVB or CHB classification. *P* values less than .05 were considered statistically significant.

## Results

After excluding 7 patients who had the pacemaker implantation at baseline and 16 at predischarge, a total of 188 consecutive patients who underwent elective transfemoral TAVR between July 16, 2020, and December 31, 2021, were enrolled. No patients withdrew from the study within the 30-day follow-up. The mean age of the patients was 73.1 (SD 7.3) years, of whom 105 (55.9%) were men, and the mean discharge days after TAVR was 2.0 (SD 1.8) days. The baseline prevalence of atrial fibrillation or flutter seemed to be higher in the group at higher risk of HAVB or CHB compared to the group at lower risk of HAVB or CHB, without statistical significance (n=15, 18.1% vs n=9, 8.6%; *P*=.05). The baseline and procedural characteristics of the study population are presented in Table 1.

**Figure 1:**
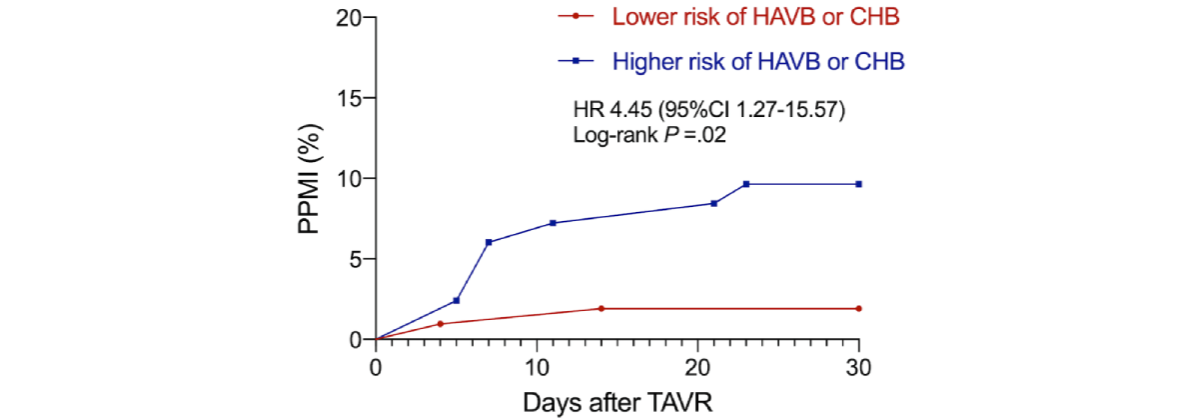
Kaplan-Meier analysis of permanent pacemaker implantation (PPMI) after discharge between groups with higher and lower risk of high-degree atrioventricular block (HAVB) or complete heart block (CHB). HR: heart rate; TAVR: transcatheter aortic valve replacement.

Two patients died within the 30-day follow-up in the group with higher HAVB or CHB risk. The patients at higher risk of HAVB or CHB received more pacemaker implantation after discharge within the 30-day follow-up (n=8, 9.6% vs n=2, 1.9%; *P*=.04). The Kaplan-Meier analysis of pacemaker implantation after discharge between the two groups is shown in Figure 1. Up to 80% of patients received the pacemaker implantation within 14 days after TAVR, and the mean time to pacemaker implantation after TAVR was similar between these two groups (mean 10.8, SD 7.2 days vs mean 9.0, SD 7.1 days; *P*=.71). For the arrhythmias detected by the smartwatch, the incidence of LBBB was higher in the group at higher HAVB or CHB risk (n=47, 56.6% vs n=34, 32.4%; *P*=.001), and more persistent LBBB was also observed in the same group (n=24, 28.9% vs n=10, 9.5%; *P*=.001). The incidence of Mobitz I, Mobitz II, and third-degree atrioventricular block (AVB) in the group with a higher risk of HAVB or CHB seemed to be higher in numerical terms compared to the group with lower HAVB or CHB risk, without statistical significance (Mobitz I: n=4, 5% vs n=1, 1%; *P*=.26; Mobitz II: n=3, 3.8% vs n=1, 1%; *P*=.44; third-degree AVB: n=4, 5% vs n=1, 1%; *P*=.23). Other arrhythmias detected by the smartwatch were comparable between these two groups. No differences were observed between these two groups in echocardiographic data during the 30-day follow-up. All outcomes during the 30-day follow-up are presented in Table 2.

There were no statistically significant changes in the evolution of atrial fibrillation or atrial flutter, Mobitz I, Mobitz II, and third-degree AVB during the first month after TAVR. The incidence of LBBB increased in the first week and decreased in the subsequent 3 weeks significantly (*P*<.001). Consistently, the QRS duration increased in the first week and decreased significantly afterward (*P*<.001). Moreover, the incidence of LBBB was higher in the higher-risk HAVB or CHB group when compared with the lower-risk group at any given time. The detailed information about different arrhythmias over time in the two groups is shown in Table S1 in Multimedia Appendix 1. The evolution of arrhythmias during the first 7 days and the first 4 weeks after TAVR is presented in Figure S1 in Multimedia Appendix 1, and the incidences of arrhythmias in total, in the higher-risk and lower-risk HAVB or CHB groups are presented in Figure S2 in Multimedia Appendix 1.

The predictors for pacemaker implantation after discharge were baseline right bundle branch block (RBBB), higher risk of HAVB or CHB, Mobitz II, and third-degree AVB detected by smartwatch in the univariate analysis. The independent predictors after discharge for pacemaker implantation were age, baseline atrial fibrillation, baseline RBBB, Mobitz II, and third-degree AVB detected by the smartwatch. The Cox regression model for pacemaker implantation after discharge within the 30-day follow-up is presented in Table 3.

**Table 1:** Baseline, electrocardiographic, imaging, and procedural characteristics of the study population, according to the high-degree atrioventricular block (HAVB) or complete heart block (CHB) risk.

Characteristics		Overall (N=188)		Higher risk of HAVB or CHB^a^ (n=83)	Lower risk of HAVB or CHB (n=105)		*P* value
Age (years), mean (SD)		73.1 (7.3)		73.9 (8.1)	72.4 (6.5)		.17
Gender (male), n (%)		105 (55.9)		46 (55.4)	59 (56.2)		.92
BMI (kg/m^2^), mean (SD)		23.40 (3.57)		23.63 (3.62)	23.21 (3.53)		.43^b^
STS^c^ (%), mean (SD)		3.78 (3.07)		4.30 (3.62)	3.40 (2.52)		.13^b^
Hypertension, n (%)		95 (51.6)		45 (54.2)	50 (49.5)		.52
Diabetes mellitus, n (%)		38 (20.4)		20 (24.1)	18 (17.5)		.27
Prior stroke, n (%)		7 (3.7)		5 (6)	2 (1.9)		.27
Atrial fibrillation or flutter, n (%)		24 (12.8)		15 (18.1)	9 (8.6)		.05
**Echocardiographic** **and computed tomographic** **variables** **, mean (SD)**							
	LVEF^d^ (%)	58.8 (11.2)	58.0 (12.2)		59.4 (10.4)	.51^b^	
	Max velocity (ms)	4.80 (0.74)	4.86 (0.81)		4.76 (0.67)	.50^b^	
	Mean gradient (mmHg)	54.8 (18.5)	55.9 (20.1)		53.9 (17.2)	.66^b^	
	AVA^e^ (cm^2^)	0.66 (0.23)	0.62 (0.24)		0.70 (0.22)	.01^b^	
	Perimeter derived diameter (mm)	25 (4)	25.1 (5.4)		24.9 (2.4)	.65	
**Electrocardiographic variables, n (%)**							
	Preexisting atrial fibrillation	23 (12.6)	12 (15)		11 (10.7)	.38	
	Preexisting RBBB^f^	13 (7.1)	7 (8.6)		6 (5.8)	.46	
	Preexisting LBBB^g^	9 (4.9)	7 (8.6)		2 (1.9)	.08	
	Increased PR or QRS interval ≥20 ms	112 (62.9)	56 (73.7)		56 (54.6)	.01	
	PR ≥240 ms or QRS ≥150 ms^h^	69 (36.7)	42 (50.6)		27 (25.7)	<.001	
**Periprocedural characteristics**							
	Predilatation, n (%)	171 (93.4)	74 (91.4)		97 (95.1)	.31	
	Postdilatation, n (%)	120 (65.9)	52 (65)		68 (66.7)	.81	
	Self-expanding valve, n (%)	160 (88.4)	71 (88.8)		89 (88.1)	.90	
	Prosthetic valve size >26mm, n (%)	134 (74)	56 (70)		78 (77.2)	.27	
	Oversizing ratio, mean (SD)	1.06 (0.09)	1.06 (0.11)		1.06 (0.07)	.69	
Days discharged after TAVR^i^ (days), mean (SD)		2 (1.8)		1 (0)	3.3 (2.1)		<.001^b^

^a^Higher risk of HAVB or CHB was defined by our conduction management protocol in electrocardiogram (supplementary methods in Multimedia Appendix 1).

^b^Mann-Whitney *U* test was used.

^c^STS: Society of Thoracic Surgeons.

^d^LVEF: left ventricular ejection fraction.

^e^AVA: aortic valve area.

^f^RBBB: right bundle branch block.

^g^LBBB: left bundle branch block.

^h^QRS ≥140 ms for patients with atrial fibrillation.

^i^TAVR: transcatheter aortic valve replacement.

**Table 2:** thirty days outcomes of the study population, according to the high-degree atrioventricular block (HAVB) or complete heart block (CHB) risk group.

Characteristics				Overall (N=188)		Higher risk of HAVB or CHB^a^ (n=83)		Lower risk of HAVB or CHB (n=105)		*P* value
Mortality, n (%)				2 (1.1)		2 (2.4)		0 (0)		.19
PPMI^b^ after discharge, n (%)				10 (5.3)		8 (9.6)		2 (1.9)		.04
Time to PPMI since TAVR^c^, mean (SD)				10.4 (6.8)		10.8 (7.2)		9.0 (7.1)		.60^d^
PPMI within 14 days since TAVR, n (%)				8 (80)		6 (75)		2 (100)		>.99
**Arrhythmias detected by smartwatch, n (%)**										
	**Bradyarrhythmia**			84 (44.7)		48 (57.8)		36 (34.3)		.002
		LBBB^e^	81 (43.1)		47 (56.6)		34 (32.4)		.001	
		Persistent LBBB	34 (18.1)		24 (28.9)		10 (9.5)		.001	
		Mobitz I	5 (2.7)		4 (5)		1 (1)		.23	
		Mobitz II	4 (2.2)		3 (3.8)		1 (1)		.44	
		Third AVB^f^	5 (2.7)		4 (5)		1 (1)		.23	
	**Tachyarrhythmia**			33 (17.9)		17 (21.3)		16 (15.4)		.30
		Atrial fibrillation or atrial flutter	33 (17.9)		17 (21.3)		16 (15.4)		.30	
		ventricular tachycardia	0 (0)		0 (0)		0 (0)		—^g^	
		PVST^h^	0 (0)		0 (0)		0 (0)		—	
	Significant pause (>5 s)			7 (4.1)		4 (5.4)		3 (3.1)		.72
**Echocardiographic data, mean (SD)**										
	LVEF^i^ (%)			60.5 (9.4)		58.9 (11.8)		61.5 (7.3)		.57^d^
	Max velocity (ms)			2.31 (0.51)		2.30 (0.50)		2.31 (0.52)		.81^d^
	Mean gradient (mmHg)			11.3 (5.1)		11.3 (4.6)		11.3 (5.4)		.83^d^
	AVA^j^ (cm^2^)			1.67 (0.43)		1.66 (0.45)		1.68 (0.42)		.42^d^

^a^Higher risk of HAVB/CHB was defined by our conduction management protocol in electrocardiogram (supplementary methods in Multimedia Appendix 1).

^b^PPMI: pacemaker implantation.

^c^TAVR: transcatheter aortic valve replacement.

^d^Mann-Whitney *U* test was used.

^e^LBBB: left bundle branch block.

^f^AVB: atrioventricular block.

^g^Not applicable.

^h^PVST: paroxysmal supraventricular tachycardia.

^i^LVEF: left ventricular ejection fraction.

^j^AVA: aortic valve area.

**Table 3:** Cox regression models for pacemaker implantation after discharge within 30-day follow-up.

Variables before discharge	Univariate regression		Multivariate regression	
	β (SE)	*P* value	β (SE)	*P* value
Age	0.023 (0.044)	.61	–0.106 (0.047)	.03
Gender (male)	0.621 (0.690)	.37	—^a^	—
Baseline atrial flutter or atrial fibrillation	1.128 (0.690)	.10	2.923 (0.999)	.003
Baseline RBBB^b^	1.821 (0.691)	.01	3.395 (1.079)	.002
Baseline LBBB^c^	0.832 (1.054)	.43	—	—
Predilatation	0.500 (1.054)	.64	—	—
Self-expanding valve	0.663 (0.791)	.40	—	—
Oversizing ratio	1.421 (2.913)	.63	—	—
Postdilatation	1.102 (0.646)	.09	—	—
Higher risk of HAVB^d^ or CHB^e^	1.648 (0.791)	.04	—	—
Atrial fibrillation or atrial flutter^f^	1.168 (0.646)	.07	—	—
LBBB^f^	0.272 (0.632)	.67	—	—
Mobitz I^f^	1.445 (1.054)	.17	—	—
Mobitz II^f^	3.155 (0.697)	<.001	4.523 (1.160)	<.001
Third AVB^f,g^	2.849 (0.693)	<.001	2.898 (0.971)	.003

^a^Not applicable.

^b^RBBB: right bundle branch block.

^c^LBBB: left bundle branch block.

^d^HAVB: high-degree atrioventricular block.

^e^CHB: complete heart block. Higher risk of HAVB or CHB was defined by our conduction management protocol in electrocardiogram supplementary methods in Multimedia Appendix 1).

^f^Arrhythmias after discharge detected by the smartwatch.

^g^AVB: atrioventricular block.

## Discussion

### Principal Findings

The main findings of this study are summarized as follows: (1) during the 30-day follow-up, patients at higher risk of HAVB or CHB received more pacemaker implantation after discharge compared to patients at lower risk of HAVB or CHB who were eligible for next-day discharge; (2) the incidence of LBBB was higher in patients at higher risk of HAVB or CHB while the incidence of other arrhythmias and CDs seemed similar in these two groups; (3) the age, baseline atrial fibrillation, baseline RBBB, Mobitz II, and third-degree AVB detected by the smartwatch were independent predictors for pacemaker implantations after discharge from the index TAVR hospitalization.

Data from the Healthcare Cost and Utilization Project Nationwide Readmission Database reported an increase from 5.7% to 13% in the proportion of pacemaker implantation in patients receiving TAVR after the promotion of minimalist TAVR and early discharge strategies in recent years [8]. The increasing trend of pacemaker implantation was likely to be driven by the reduced length of stay after TAVR, and hence, a risk stratification and monitoring protocol was urgently needed. However, the data using the ambulatory ECG monitoring to observe the proportion of pacemaker implantation were scarce. In their study, Tian et al [9] performed a remote monitoring system—BodyGuardian (Preventice Technologies)—in patients who received the TAVR procedure and found within 30 days after discharge, 8.7% of patients received pacemaker implantation, of which 7.1% were for symptomatic second or third-degree AVB and 1.6% were for symptomatic sinus node dysfunction after discharge within the 30-day follow-up [9]. A recent study by Muntane-Carol et al [3] also performed the systematic 2-week ambulatory ECG monitoring (CardioSTAT [Icentia] or Zio AT Patch [iRhythm Technologies]) following minimalist TAVR and detected an incidence of 2.2% for pacemaker implantation in the group with no ECG changes, an incidence of 6.6% in the new ECG CD group, and an incidence of 13.2% in the baseline RBBB group [3]. Similarly, Okoh et al [10] observed an incidence of 0%, 4%, and 8.5% for pacemaker implantation in group I (normal pre-TAVR, periprocedural, and discharge ECGs), group II (normal pre-TAVR and abnormal subsequent ECGs), and group III (abnormal baseline and abnormal subsequent ECGs), respectively, with a real-time home continuous ECG system (Zio AT Patch [iRhythm Technologies]). The overall incidence of pacemaker implantation in our study was 5.3%, while the incidence in the higher-risk HAVB or CHB group was up to 9.6%. This was similar to previous studies with ambulatory ECG monitoring after TAVR. Our data also showed that 80% of pacemakers were implanted within 14 days after TAVR, similar to the results reported by Mazzella et al [8]. The rate of pacemaker implantation after discharge was similar in these studies, whether or not ambulatory ECGs were used. However, the rate increased in the specific group of patients with a higher risk of HAVB or CHB according to the modified protocol in our study (similar to the new ECG CDs group or baseline RBBB group in the study by Muntane-Carol et al [3]. Therefore, we can draw the inference that ambulatory ECG use does not increase the rate of pacemaker implantation after discharge, but it can monitor the arrhythmias or CDs in higher-risk patients who may be more likely to receive the pacemaker implantation on the discharging day. However, this conclusion should be interpreted cautiously, as our study did not compare the incidence of arrhythmic events monitored with or without smartwatches.

In a recent study by Reiter et al [4], 59 consecutive patients who received implantable loop recorders were enrolled; an LBBB incidence of 28.8% on the first day after TAVR, 32.2% on the second day, and 33.9% on the third day were observed, while the incidence decreased to 25.9% at the first-month follow-up. The incidence of LBBB in the above-mentioned study was higher than that in our study, which might be due to the higher oversizing ratio of the implanted prosthetic valve. Consistent with our previous pilot research data, the prevalence of LBBB increased within the first week after TAVR and decreased in the subsequent weeks following the TAVR, which was in line with the resolution of edema and inflammation in the area of the His bundle and the left bundle branch [11]. The prevalence of LBBB was up to 39.4% in the whole cohort, while the prevalence was 51.8% in patients at a higher risk of HAVB or CHB. Urena et al [12] and Muntane-Carol et al [13] reported that persistent LBBB may result in more HAVB or CHB episodes requiring pacemaker implantation. In our study, only 2 of the 10 patients requiring pacemaker implantation developed new-onset persistent LBBB, while 3 had transient LBBB (with 2 patients developing the LBBB on the first day after TAVR and 1 developing it on the third day). Moreover, in our study, we observed a higher prevalence of LBBB in patients at risk of HAVB or CHB. Nearly half a percentage of LBBB was persistent LBBB in this group, while only one-third of LBBB was persistent in patients eligible for next-day discharge.

Of the 44.1% of patients classified as being at higher risk of HAVB or CHB, 9.6% ultimately received the pacemaker implantation after discharge within the 30-day follow-up. Most of the pacemakers were implanted within 14 days after the TAVR procedure. However, 2 patients in the group with a higher risk of HAVB or CHB received the pacemaker implantation on the 21st and 23rd days post TAVR for symptomatic significant pause (atrial flutter and LBBB in the previous single ECG) and delayed HAVB, respectively. Our study indicated that the intensive remote monitoring of arrhythmias and CDs within the first 14 days after TAVR was very necessary, and the monitor continued to play a critical role in the subsequent days of the first months. It was reasonable that the Mobitz II and third-degree AVB detected by the smartwatch were independent predictors of pacemaker implantation. Additionally, age, baseline atrial fibrillation, and baseline RBBB were independent predictors for pacemaker implantation. However, the study did not observe baseline LBBB, self-expanding valve, and prosthesis oversizing ratio as independent predictors of pacemaker implantation after discharge as most commonly reported [14]. Although clinical judgment evaluating patients at a higher risk of HAVB or CHB was recommended by expert consensus, it was still challenging to identify this population [2]. In our previous case, 1 patient who was eligible for next-day discharge received the pacemaker implantation after discharge for symptomatic delayed HAVB detected by the smartwatch [15]. Therefore, additional strategies for refining the risk assessment are warranted [2]. Our strategy of arrhythmias monitored by a smartwatch during follow-ups may provide more information, proving to be a better predictor for pacemaker implantation after discharge.

Although a significant portion of the technology is still in its nascent stages, encompassing numerous technical challenges, such as data handling, analysis, storage, security, and privacy, it is imperative to foster collaboration between the health and technical domains, involving a diverse range of experts with varied skills and domain knowledge [6]. Engaging health care professionals in smart technology research will not only ensure the relevance of these tools but also enhance their successful implementation.

### Study Limitations

First, this was a single-center, nonrandomized cohort study. There may be some bias since nearly all enrolled patients were in good medical compliance, while 1 patient with asymptomatic HAVB declined the pacemaker implantation. The SMART TAVR study involved patients who had undergone a TAVR procedure, which likely contributed to higher compliance levels, driven by their postprocedural monitoring and psychological care requirements [5]. Moreover, the small number of TAVR recipients with balloon-expandable prosthetic valves (11.6%) may interfere with the results. Additionally, numerous ongoing studies are currently investigating the use of smart wearable devices for monitoring atrial fibrillation. There is a noticeable gap in research regarding the detection of bradyarrhythmia. Finally, the limited number of pacemaker implantation cases may influence the independent predictor evaluation.

### Conclusions

Except for LBBB, no change in arrhythmias was observed over time in the first month after TAVR. Patients at a high risk of HAVB or CHB received more pacemaker implantation after discharge. More prevalence of LBBB was observed in the HAVB or CHB group compared with the patients who were eligible for next-day discharge while the other arrhythmias were similar between these two groups. Smartwatch-monitored arrhythmias during follow-up were valuable factors in predicting pacemaker implantation after discharge from the index TAVR.

## Appendix

Multimedia Appendix 1 Additional information and statistics.

## Abbreviations

AVB: atrioventricular block
CD: conduction disturbance
CHB: complete heart block
ECG: electrocardiogram
HAVB: high-degree atrioventricular block
LBBB: left bundle branch block
RBBB: right bundle branch block
SMART TAVR: smartwatch-facilitated early discharge in patients undergoing TAVR
TAVR: transcatheter aortic valve replacement

## Data Availability

The data sets generated during and/or analyzed during this study are not publicly available but are available from the corresponding author on reasonable request.
