# Supplementary material for: Smartwatch-Detected Arrhythmias in Patients After Transcatheter Aortic Valve Replacement (TAVR): Analysis of the SMART TAVR Trial
Source: J Med Internet Res. 2024 Jul 19;26:e41843. doi: 10.2196/41843 (PMC11297386; doi:10.2196/41843)
Supplement: Multimedia Appendix 1 [file jmir_v26i1e41843_app1.docx]

**SUPPLEMENTARY MATERIAL**

Smartwatch-Detected Arrhythmias in Patients After Transcatheter Aortic Valve Replacement (TAVR): Analysis of the SMART TAVR Trial

**Supplementary Method**

**Group 1. No ECG changes in patients without pre-procedural high-risk factors for conduction block***

- If no ECG changes immediately procedure and 4 hours after procedure, remove temporary pacemaker at the end of procedure or within 4 hours, continuous telemetry for 24 hours and perform 12-lead ECG at 24 hours after procedure.
- If no ECG changes at 24 hours, the patient can be discharged at 1 day after procedure.
- If there are new-onset atrioventricular block (AVB, RBBB, LBBB, QRS≥120 ms) or ECG changes (PR or QRS interval prolong≥20 ms) or bradyarrhythmia at 24 hours after procedure, refer to **Group 3**.
- Proceed with permanent pacemaker implantation if persistent HAVB/CHB*.

**Group 2. No ECG changes in patients with pre-procedural high-risk factors for conduction block***

- If no ECG changes immediately procedure and 4 hours after procedure, reserve temporary pacemaker for 24 hours post-TAVR, continuous telemetry for 24 hours and perform 12-lead ECG at 24 hours after procedure.
- If no ECG changes at 24 hours, remove temporary pacemaker and the patient can be discharge at 1 day after procedure.
- If there are new-onset atrioventricular block (AVB, RBBB, LBBB, QRS≥120 ms) or ECG changes (PR or QRS interval prolong≥20 ms) or bradyarrhythmia at 24 hours after procedure, refer to **Group 3**.
- Proceed with permanent pacemaker implantation if persistent HAVB/CHB*.

**Group 3. ECG changes during or post-TAVR in patients**

- If there are new-onset atrioventricular block (AVB, RBBB, LBBB, QRS≥120 ms) or ECG changes (PR or QRS interval prolong≥20 ms) or bradyarrhythmia during or post-TAVR immediately procedure and 4 hours after procedure, reserve temporary pacemaker and continuous telemetry for 24 hours and perform 12-lead ECG at 24 hours after procedure.
- If ECG changes regress (to baseline values, irrespective of QRS/PR interval duration) or no further ECG changes with QRS ≤150 ms (for atrial fibrillation QRS ≤140 ms) and PR ≤240 ms at 24 hours, remove temporary pacemaker and discharge at 1 day after procedure.
- If there are further ECG changes (persistent increase of ≥20 ms in the PR or QRS interval) at 24 hours, reserve temporary pacemaker and continuous telemetry for another 24 hours, perform holter electrocardiogram/electrophysiological evaluation and daily 12-lead ECG after procedure.
- Proceed with permanent pacemaker implantation if persistent HAVB/CHB*.

**Group 4. Transient HAVB/CHB peri-procedurally in patients**

- If transient HAVB/CHB develops during or post-procedurally, reserve temporary pacemaker, continuous telemetry perform holter electrocardiogram/electrophysiological evaluation and daily 12-lead ECG after procedure.
- If no further ECG changes (persistent increase of ≥20 ms in the PR or QRS interval) , no recurrent HAVB/CHB and no evidence of HAVB/CHB or long RR intervals (no need for permanent pacemaker implantation) in holter electrocardiogram, remove temporary pacemaker, continuous telemetry and daily ECG, and the patient can be discharged after ECG stable for 24 hours.
- Proceed with permanent pacemaker implantation if persistent HAVB/CHB*.

* High risk factors evaluated by pre-TAVR electrocardiogram: left bundle branch block, right bundle branch block, ventricular block (QRS≥120ms or 1° atrioventricular block) high-grade atrioventricular block. High risk factors evaluated by pre-TAVR CT: membranous septum < 2.5mm, left ventricular outflow tract calcification (landing zone calcification).

**Table S1. Arrhythmias over time in total, higher risk, and lower risk of HAVB or CHB groups**

(A). Af over time in total, higher risk, and lower risk of HAVB or CHB groups

| Af | Total n = 188 | Higher risk n = 83 | Lower risk n = 105 | p value |
| --- | --- | --- | --- | --- |
| +1 | 21(11.2) | 12(14.5) | 9(8.6) | 0.203 |
| +2 | 22(11.8) | 12(14.6) | 10(9.5) | 0.282 |
| +3 | 23(12.4) | 12(14.6) | 11(10.6) | 0.404 |
| +4 | 22(11.7) | 12(14.5) | 10(9.5) | 0.296 |
| +5 | 23(12.3) | 12(14.6) | 11(10.5) | 0.390 |
| +6 | 23(12.2) | 12(14.5) | 11(10.5) | 0.408 |
| +7 | 23(12.2) | 12(14.5) | 11(10.5) | 0.408 |
| 2w | 24(12.8) | 13(15.9) | 11(10.5) | 0.275 |
| 3w | 23(12.3) | 14(17.1) | 9(8.6) | 0.079 |
| 4w | 22(11.8) | 12(14.8) | 10(9.5) | 0.268 |
| p value | 1.000 | 1.000 | 1.000 |  |

(B). LBBB over time in total, higher risk, and lower risk of HAVB or CHB groups

| LBBB | Total n = 188 | Higher risk n = 83 | Lower risk n = 105 | p value |
| --- | --- | --- | --- | --- |
| +1 | 45(23.9) | 30(36.1) | 15(14.3) | <0.001 |
| +2 | 53(28.3) | 34(41.5) | 19(18.1) | <0.001 |
| +3 | 60(32.3) | 38(46.3) | 22(21.2) | <0.001 |
| +4 | 71(37.8) | 42(50.6) | 29(27.6) | 0.001 |
| +5 | 71(38.0) | 43(52.4) | 28(26.7) | <0.001 |
| +6 | 74(39.4) | 43(51.8) | 31(29.5) | 0.002 |
| +7 | 71(37.8) | 41(49.4) | 30(28.6) | 0.003 |
| 2w | 68(36.4) | 43(52.4) | 25(23.8) | <0.001 |
| 3w | 44(23.5) | 32(39.0) | 12(11.4) | <0.001 |
| 4w | 37(19.9) | 26(32.1) | 11(10.5) | <0.001 |
| p value | <0.001 | 0.051 | <0.001 |  |

(C). Mobitz I over time in total, higher risk, and lower risk of HAVB or CHB groups

| Mobitz I | Total n = 188 | Higher risk n = 83 | Lower risk n = 105 | p value |
| --- | --- | --- | --- | --- |
| +1 | 1(0.5) | 1(1.2) | 0(0.0) | 0.441 |
| +2 | 2(1.1) | 2(2.4) | 0(0.0) | 0.191 |
| +3 | 2(1.1) | 1(1.2) | 1(1.0) | 1.000 |
| +4 | 2(1.1) | 2(2.4) | 0(0.0) | 0.194 |
| +5 | 1(0.5) | 1(1.2) | 0(0.0) | 0.439 |
| +6 | 1(0.5) | 1(1.2) | 0(0.0) | 0.441 |
| +7 | 2(1.1) | 2(2.4) | 0(0.0) | 0.194 |
| 2w | 2(1.1) | 2(2.4) | 0(0.0) | 0.191 |
| 3w | 2(1.1) | 2(2.4) | 0(0.0) | 0.191 |
| 4w | 3(1.6) | 3(3.7) | 0(0.0) | 0.161 |
| p value | 0.990 | 0.980 | 0.865 |  |

(D). Mobitz II over time in total, higher risk, and lower risk of HAVB or CHB groups

| Mobitz II | Total n = 188 | Higher risk n = 83 | Lower risk n = 105 | p value |
| --- | --- | --- | --- | --- |
| +1 | 0(0.0) | 0(0.0) | 0(0.0) | - |
| +2 | 0(0.0) | 0(0.0) | 0(0.0) | - |
| +3 | 0(0.0) | 0(0.0) | 0(0.0) | - |
| +4 | 2(1.1) | 1(1.2) | 1(1.0) | 1.000 |
| +5 | 1(0.5) | 1(1.2) | 0(0.0) | 0.439 |
| +6 | 0(0.0) | 0(0.0) | 0(0.0) | - |
| +7 | 0(0.0) | 0(0.0) | 0(0.0) | - |
| 2w | 3(1.6) | 3(3.7) | 0(0.0) | 0.165 |
| 3w | 1(0.5) | 1(1.2) | 0(0.0) | 0.439 |
| 4w | 1(0.5) | 1(1.2) | 0(0.0) | 0.435 |
| p value | 0.163 | 0.229 | 0.867 |  |

(E). Third degree AVB over time in total, higher risk, and lower risk of HAVB or CHB groups

| Third AVB | Total n = 188 | Higher risk n = 83 | Lower risk n = 105 | p value |
| --- | --- | --- | --- | --- |
| +1 | 1(0.5) | 1(1.2) | 0(0.0) | 0.441 |
| +2 | 2(1.1) | 2(2.4) | 0(0.0) | 0.191 |
| +3 | 2(1.1) | 2(2.4) | 0(0.0) | 0.193 |
| +4 | 2(1.1) | 2(2.4) | 0(0.0) | 0.194 |
| +5 | 2(1.1) | 2(2.4) | 0(0.0) | 0.191 |
| +6 | 3(1.6) | 2(2.4) | 1(1.0) | 0.429 |
| +7 | 3(1.6) | 2(2.4) | 1(1.0) | 0.429 |
| 2w | 1(0.5) | 1(1.2) | 0(0.0) | 0.439 |
| 3w | 0(0.0) | 0(0.0) | 0(0.0) | - |
| 4w | 1(0.5) | 1(1.2) | 0(0.0) | 0.435 |
| p value | 0.853 | 0.876 | 0.694 |  |

(F). PR interval over time in total, higher risk, and lower risk of HAVB or CHB groups

| PR | Total n = 188 | Higher risk n = 83 | Lower risk n = 105 | p value |
| --- | --- | --- | --- | --- |
| +1 | 170.3±34.0 | 174.6±39.7 | 161.1±21.2 | 0.078* |
| +2 | 172.7±37.1 | 180.7±44.2 | 164.5±24.2 | 0.064* |
| +3 | 175.3±41.3 | 186.8±50.7 | 165.3±28.4 | 0.014* |
| +4 | 174.5±39.9 | 185.0±42.3 | 164.9±33.2 | 0.002* |
| +5 | 179.3±43.9 | 190.6±49.3 | 169.3±35.7 | 0.012* |
| +6 | 181.5±41.9 | 192.9±50.1 | 171.2±30.6 | 0.010* |
| +7 | 180.1±40.8 | 191.2±48.6 | 170.7±30.9 | 0.029* |
| 2w | 180.0±42.6 | 192.8±52.3 | 169.3±32.1 | 0.011* |
| 3w | 178.5±40.3 | 190.2±48.5 | 169.3±30.6 | 0.005* |
| 4w | 175.1±41.8 | 186.2±53.1 | 166.2±30.0 | 0.025* |
| p value | 0.116* | 0.503* | 0.503* |  |

(G). QRS duration over time in total, higher risk, and lower risk of HAVB or CHB groups

| QRS | Total n = 188 | Higher risk n = 83 | Lower risk n = 105 | p value |
| --- | --- | --- | --- | --- |
| +1 | 121.3±35.3 | 128.5±39.2 | 109.7±28.0 | <0.001* |
| +2 | 124.6±35.0 | 131.9±36.4 | 111.7±29.6 | <0.001* |
| +3 | 128.3±34.9 | 136.6±36.9 | 115.9±30.0 | <0.001* |
| +4 | 130.7±36.3 | 138.6±36.4 | 118.6±33.0 | <0.001* |
| +5 | 132.0±37.4 | 141.6±37.6 | 118.2±33.0 | <0.001* |
| +6 | 133.0±37.0 | 141.1±36.4 | 120.1±33.8 | <0.001* |
| +7 | 131.7±37.2 | 140.0±37.9 | 119.0±32.7 | <0.001* |
| 2w | 130.1±35.3 | 139.2±36.5 | 117.6±30.6 | <0.001* |
| 3w | 122.4±34.0 | 129.0±35.8 | 109.9±26.6 | <0.001* |
| 4w | 119.6±33.9 | 124.0±36.2 | 108.7±25.8 | 0.001* |
| p value | <0.001* | 0.029* | 0.051* |  |

*Kruskal-Wallis Test was used.

**Figure S1. Evolution of Arrhythmias after TAVR**


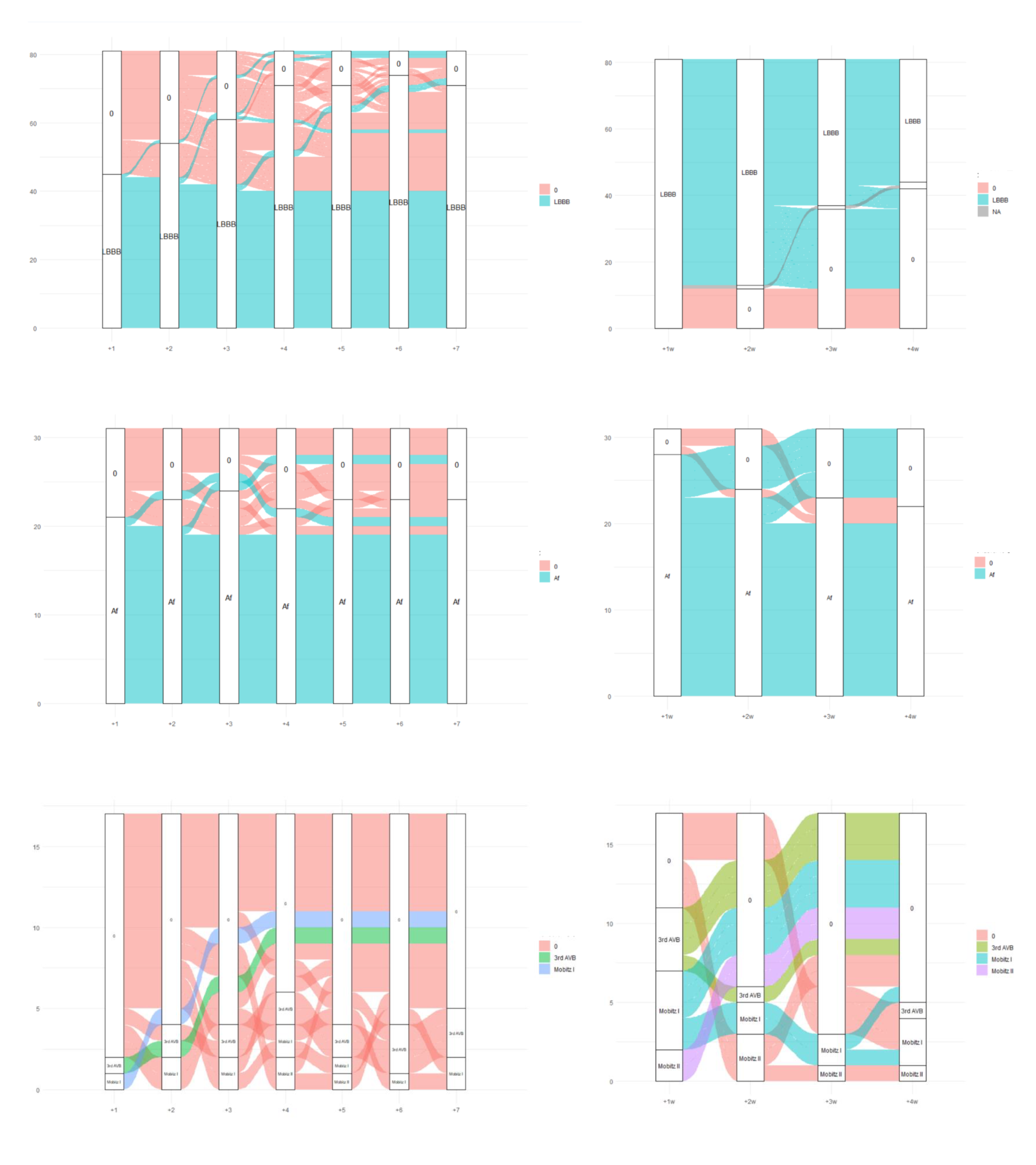


**Figure S2. Incidence of Arrhythmias after TAVR**

**
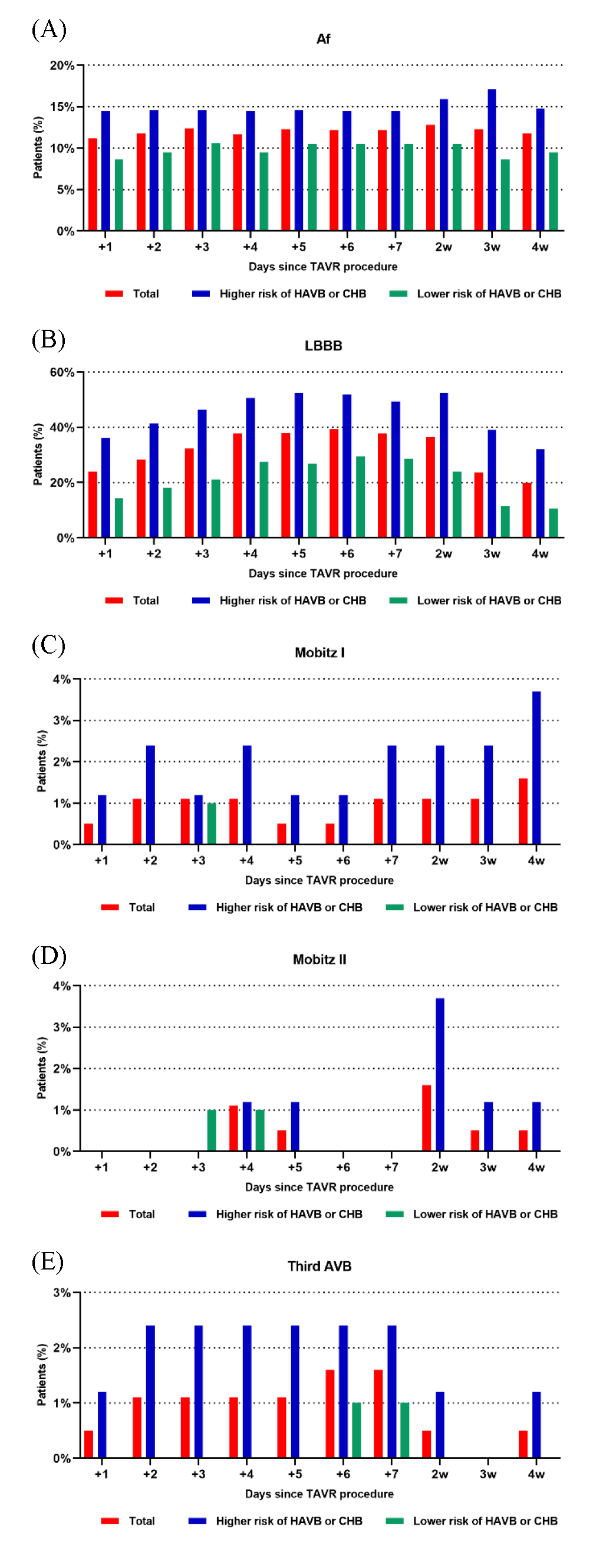
**
